# Management of Oxidative Stress: Crosstalk Between Brown/Beige Adipose Tissues and Skeletal Muscles

**DOI:** 10.3389/fphys.2021.712372

**Published:** 2021-09-16

**Authors:** Ruping Pan, Yong Chen

**Affiliations:** ^1^Department of Nuclear Medicine, Tongji Hospital, Tongji Medical College, Huazhong University of Science and Technology, Wuhan, China; ^2^Department of Endocrinology, Internal Medicine, Tongji Hospital, Tongji Medical College, Huazhong University of Science and Technology, Wuhan, China; ^3^Branch of National Clinical Research Center for Metabolic Diseases, Wuhan, China

**Keywords:** exercise, oxidative stress, brown adipose tissue, beige adipose tissue, crosstalk, skeletal muscle

## Abstract

Exercise plays an important role in the physiology, often depending on its intensity, duration, and frequency. It increases the production of reactive oxygen species (ROS). Meanwhile, it also increases antioxidant enzymes involved in the oxidative damage defense. Prolonged, acute, or strenuous exercise often leads to an increased radical production and a subsequent oxidative stress in the skeletal muscles, while chronic regular or moderate exercise results in a decrease in oxidative stress. Notably, under pathological state, such as obesity, aging, etc., ROS levels could be elevated in humans, which could be attenuated by proper exercise. Significantly, exercise stimulates the development of beige adipose tissue and potentially influence the function of brown adipose tissue (BAT), which is known to be conducive to a metabolic balance through non-shivering thermogenesis (NST) and may protect from oxidative stress. Exercise-related balance of the ROS levels is associated with a healthy metabolism in humans. In this review, we summarize the integrated effects of exercise on oxidative metabolism, and especially focus on the role of brown and beige adipose tissues in this process, providing more evidence and knowledge for a better management of exercise-induced oxidative stress.

## Introduction

Physical activity benefits physiology in humans in many aspects, protecting from metabolic diseases including obesity, type 2 diabetes, cardiovascular diseases, and neuropsychiatric disorders, such as Alzheimer’s disease, depression, Parkinson’s disease, etc. Physical activity also induces the production of reactive oxygen species (ROS; Thirupathi et al., 2020). Moderate ROS levels have been shown to play a role in physiological health, while excessive ROS levels often lead to an inadequate oxidative defense and thus cause oxidative stress. ROS-related diseases include obesity, diabetes, cardiovascular and neurodegenerative diseases, and aging. A balance of ROS levels in healthy individuals as well as a control of excessive ROS accumulation under pathological circumstances are both important. Of note, exercises benefit various diseases often depending on their modes, among which aerobic exercise, resistance training, and resistance exercise are mostly used types of exercise interventions for disease treatment (Luan et al., 2019). However, prolonged endurance exercise or short-duration, high intensity exercise has been demonstrated to increase the biomarkers of oxidative stress in both blood and skeletal muscle in humans. The management of exercise-induced oxidative stress is particularly meaningful. Superoxide dismutase (SOD) was first discovered in 1960s (McCord and Fridovich, 1969). Manganese superoxide dismutase (SOD2) has been declared to be a key antioxidant enzyme preventing superoxide accumulation in skeletal muscles after exercise (Hollander et al., 2001). Especially, exercise-induced increase in SOD2 levels protects cardiomyocytes from ischemia–reperfusion injury (Yamashita et al., 1999). Furthermore, other antioxidant enzymes such as catalase, glutathione peroxidase, glutathione reductase, glutathione-S-transferase as well as nonenzymatic antioxidants such as vitamins E, A, C, glutathione and uric acid were successively identified to be involved in the prevention of excessive exercise or aging-induced oxidative stress (Pingitore et al., 2015).

On the other hand, beige adipose tissue and brown adipose tissue (BAT) are known as thermogenic adipose tissues, contributing to energy homeostasis through non-shivering thermogenesis (NST). Though, the effect of physical exercise on BAT function appears to be controversial according to findings in rodent experiments (Xu et al., 2011; Ignacio et al., 2012; Wu et al., 2014), physical exercise induces the development of beige adipose tissue in the murine white adipose tissue (WAT), mainly in the subcutaneous depot, a process that involves the mechanism of a crosstalk between skeletal muscle and adipose tissue (Bostrom et al., 2012). It is largely mediated by myokines secreted from contracting muscles and batokines secreted from functional brown/beige adipose tissues, which affect local cells or target neighboring cells and distant organs, contributing to the whole-body metabolic homeostasis. Uncoupling protein 1 (UCP1), which is located on the mitochondrial membrane of thermogenic adipocytes, has been demonstrated to inhibit mitochondrial ROS production (Negre-Salvayre et al., 1997; Jia et al., 2019). More recently, beige adipocytes have been shown to protect from oxidative stress *via* a mechanism of iron accumulation (Min et al., 2019). In this review, we focus on the role of thermogenic adipose tissues in the management of exercise-induced oxidative stress, providing more knowledge of exercise-related oxidative stress management.

## Radical Biology and Exercise-Related Oxidative Stress

Free radicals were first reported in living cells in 1954, that they were responsible for the cell injury caused by hyperoxia and ionizing radiation (Commoner et al., 1954; Gerschman et al., 1954). Before long, free radicals were classified as one of the main causes of aging (Harman, 1956). As unsaturated electronic substances with strong oxidizability, they capture electrons from regular molecules, leading to protein architecture remodeling, and thus oxidative damage. One kind of the radicals, ROS, were found to be mainly produced by mitochondria (Chance and Williams, 1956), which is as a by-product of mitochondrial respiration. Exercise related oxidant damage were identified in the late 1970s (Dillard et al., 1978). They show that 1h of endurance exercise increases pentane production in the exhaled gas in rats, which is an index of lipid peroxidation, and its production is restrained by the administration of vitamin E. ROS were later on confirmed as common metabolites from contracting skeletal muscles during exercise, which are reduced by vitamin E (Davies et al., 1982; Jackson et al., 1985), probably because of the large content of mitochondria in the muscle cells. Thereafter, it attracts the scientists to explore the role of oxidative stressors in the whole-body metabolism, particularly, during exercises. Besides, antioxidant mechanisms have been widely investigated for countering against oxidative stress. Remarkably, in addition to some detrimental effects, ROS act as subcellular messengers in molecular signaling and are involved in the regulation of various metabolic pathways, including an induction of the antioxidant enzymes to counter oxidative stress (Thannickal and Fanburg, 2000; Peternelj and Coombes, 2011). Besides, ROS also play important roles in maintaining stem cell genomic stability, biosynthesis of certain molecules, drug detoxification, mediating the phagocytosis of the phagocytes as well as vasodilation, muscle contraction, and apoptosis (Peternelj and Coombes, 2011; Chen, 2019). Importantly, regular or moderate exercises were demonstrated to benefit cellular antioxidant management. Thus, administration of antioxidant supplements is disputed, which are supported by several studies, indicating that antioxidant supplements have a detrimental effect on the health and performance benefits of exercise training (Childs et al., 2001; McAnulty et al., 2005). The balance between ROS production and elimination determines whether they benefit or harm human bodies.

## Brown/Beige Adipose Tissues and Their Potential Roles in the Control of Oxidative Stress

Except for the issue of exercise-induced oxidative stress, physical exercise may induce the development of beige adipose tissue and may promote BAT function in mice, which have been shown to have anti-oxidative effects according to some findings (shown as follow). Besides, brown/beige adipose tissues may control skeletal muscle function through multiple mechanisms (shown as follow). Thus, a crosstalk between skeletal muscle and brown/beige adipose tissue may play a role in the control of exercise-induced oxidative stress.

### Brown and Beige Adipose Tissues

Brown adipose tissue was firmly identified as a thermogenic organ contributing to NST in 1960s (Smith and Hock, 1963; Smith, 1964). Heat generation in the tissues is attributed to the uncoupling of oxidative phosphorylation and a subsequent proton flux across the mitochondrial inner membrane, which is followed by lipolysis (Smith and Horwitz, 1969). UCP1 was identified in 1976 and termed in 1990s, which is located in BAT mitochondria and is the main regulator mediating the non-shivering thermogenesis in response to cold and adrenergic stimulation (Ricquier and Kader, 1976). Brown-like adipocytes were described as early as 1984 in mice (Young et al., 1984), and were termed beige adipocytes in 2012 (Bostrom et al., 2012). They emerge in WAT after cold or adrenergic stimulation, contain lots of UCP1-positive mitochondria and function similarly to brown adipocytes. Human BAT was discovered using ^18^F-fluorodeoxyglucose positron emission tomography/computed tomography (^18^F-FDG PET/CT) in adults at the beginning of 21st century (Cypess et al., 2009; van Marken Lichtenbelt et al., 2009; Virtanen et al., 2009). However, it remains controversial whether human BAT is composed of brown or beige adipocytes, owing to its different location from the classic BAT. The most acceptable theory is that human BAT contains both brown and beige adipocytes according to the anatomical and transcriptome profiling (Cypess et al., 2013; Jespersen et al., 2013). Studies on BAT and beige adipose tissue mainly focus on their anti-obesity function due to their thermogenic capacity when targeted. Less is known about their roles in the management of ROS production.

### Adaption of Adipose Tissues to Exercises

**Figure 1:**
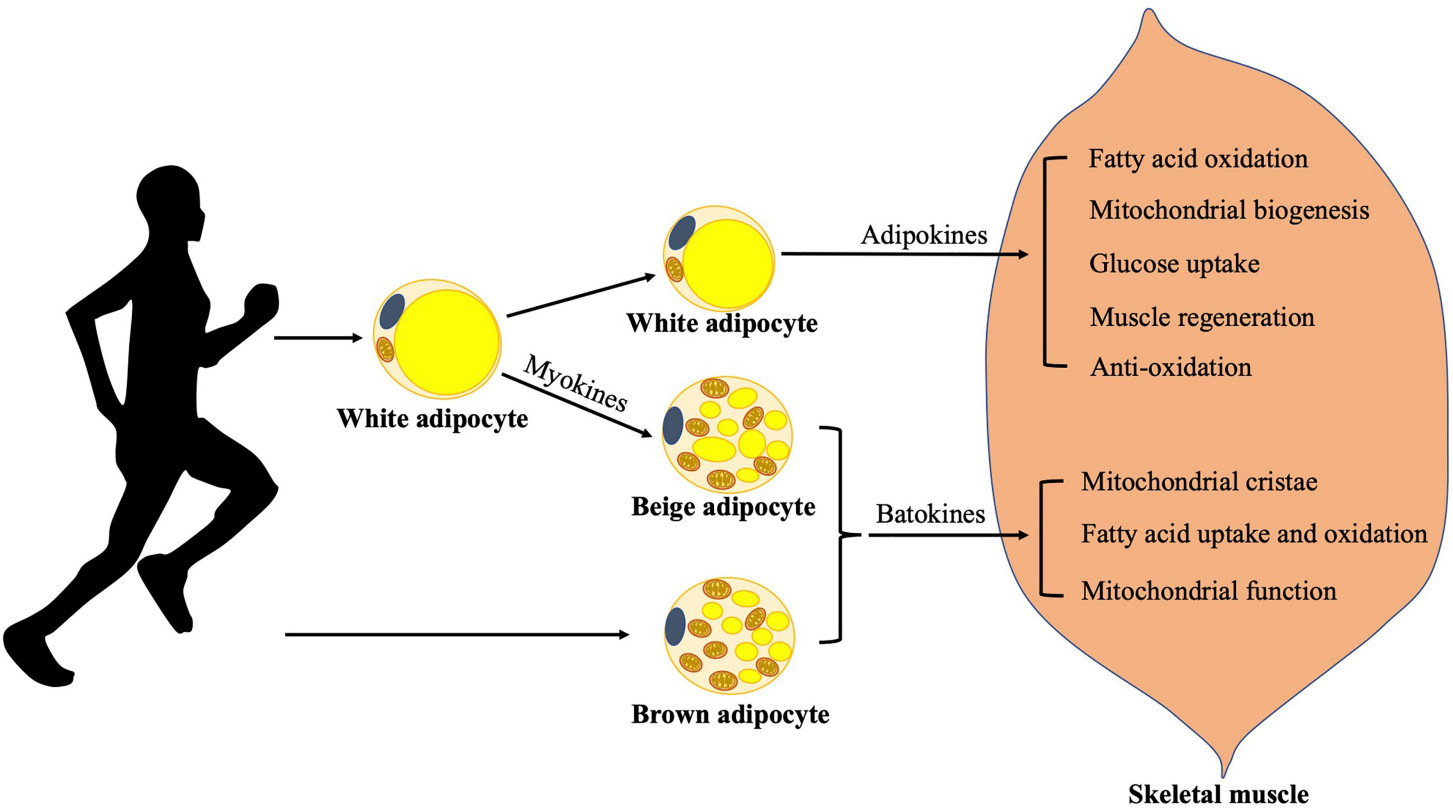
Adaption of adipose tissues to exercises. Exercises increase fat mobilization, meanwhile exercised WAT releases various adipokines benefiting skeletal muscle function in many aspects. They could induce fatty acid oxidation, mitochondrial biogenesis, glucose uptake, muscle regeneration, and display anti-oxidative effect in skeletal muscle. Contracting muscles release various myokines, which may mediate exercise-induced beige adipose tissue development and probably promotes BAT function. Batokines released from brown/beige adipose tissues may influence skeletal muscle function by promoting the formation of mitochondrial cristae, fatty acid uptake and oxidation, and mitochondrial function. BAT, brown adipose tissue and WAT, white adipose tissue.

Regular exercise training has been known to cause multiple metabolic adaptations in WAT, as shown in Figure 1, including a decrease in white adipocyte size/number and lipid content, an increase in mitochondrial biogenesis and an improvement in mitochondrial function (Stallknecht et al., 1991; Gollisch et al., 2009; Sutherland et al., 2009). Acute exercise stimulates adipose tissue blood flow and fat mobilization, resulting in delivery of fatty acids to skeletal muscles to meet their metabolic requirements (involvement of various cytokines and hormones), while chronic exercise generates an energy deficit, which results in an enhanced fat mobilization, presumably due to a superimposition of each bout of exercise, and thus a decrease in fat mass (Thompson et al., 2012). Moreover, regular exercise training also has long term effects on local or distant organs including liver, heart, and skeletal muscles (Thompson et al., 2012). Of note, transplantation of trained WAT in sedentary mice results in an improvement of glucose uptake in skeletal muscles and whole-body metabolism, possibly due to an altered adipokine profile (including adiponectin, leptin, interleukin 6, and tumor necrosis factor alpha, etc.) in WAT after training (Stanford et al., 2015). Remarkably, the expression of adiponectin receptors in WAT is significantly increased in humans after 4–12weeks exercise training (Bluher et al., 2007).

Besides, brown adipocyte markers including UCP1 and PR domain zinc finger protein 16 (PRDM16) have been found to be increased in WAT after regular training, as well as the presence of brown-like adipocytes in WAT in rodents (Bostrom et al., 2012; Stanford et al., 2015). Spiegelman group identified increased irisin levels in murine blood circulation, which is a myokine secreted by contracting muscle during exercise, drove the development of brown-like adipose tissue in WAT and thereby an increase in energy expenditure (Bostrom et al., 2012). The process is dependent on transcriptional co-activator peroxisome proliferator-activated receptor-gamma co-activator-1a (PGC-1a), which can be induced by exercise and primarily regulates UCP1, mitochondrial biogenesis, and oxidative metabolism (Puigserver et al., 1998; Handschin and Spiegelman, 2008). Furthermore, resting plasma levels of irisin have been found twice as high as baseline levels in humans after 10weeks of aerobic training through regular cycling, indicating possible regulatory effects of exercise-induced irisin on thermogenic adipose tissue development in humans as well. Besides muscle contraction during exercise, cold-induced muscle shivering has also been found to induce the secretion of irisin, which goes proportional to shivering intensity and is equivalent to exercise-induced irisin secretion in humans (Lee et al., 2014). Treatment with fibronectin type III domain containing 5 (FNDC5), which is cleaved and secreted as irisin, upregulates the expression of various thermogenic factors and increase thermogenesis in human neck adipocytes (Lee et al., 2014). However, a human study later on compared the effect of an acute endurance workload of 45min at 70% of VO_2_max and 12week intervention of combined endurance- and strength-training with four sessions of training/week on BAT metabolism (Norheim et al., 2014). Plasma levels of irisin were found acutely increased after acute exercise but reduced after chronic exercise. Moreover, no novel effect of training has been observed on beige fat development in humans according to their findings. These results are not consistent with the previous findings in rodents, indicating different responses of BAT to training in rodents and humans. Likely, there exist differences between human BAT and rodent BAT from anatomy to physiology (Pan et al., 2020). Further studies are required to better understand the effects of exercise on BAT biology in humans.

Other exercise-induced myokines, such as fibroblast growth factor 21 (FGF21), brain-derived neurotrophic factor (BDNF), vascular endothelial growth factor (VEGF), adiponectin, leptin, β-Aminoisobutyric acid, follistatin, meteorin-like, interleukin-6, lactate, and succinate have been described to show a stimulative effect on thermogenic gene expression in different cell types or WAT, either inducing WAT browning or promoting BAT function (Braga et al., 2014; Carriere et al., 2014; Knudsen et al., 2014; Rao et al., 2014; Vishvanath et al., 2016; Mills et al., 2018), while myostatin plays a negative role in beige adipose tissue development (Ruiz de Azua et al., 2017; Becerril et al., 2019), whose activity can be suppressed by decorin, another contraction-induced myokine (Kanzleiter et al., 2014). The above regulatory roles of myokines in brown/beige adipose tissue biology are well summarized by several groups (Mendez-Gutierrez et al., 2020; Rodriguez et al., 2020b). Some but not all of the above myokines have been demonstrated to have regulatory effects in humans (Mendez-Gutierrez et al., 2020). Myokines therefore may be the main causes mediating exercise-induced effects on thermogenic adipose tissues. However, their roles in humans need to be further investigated.

The results about the influence of exercises on BAT function seem to be controversial as well according to findings in rodents. Some showed positive effects of 6–8weeks of swimming or treadmill training on BAT biology with increased enzyme activity of type 2 deiodinase (DIO2), mitochondrial activity, and respiration, BAT activity and the expression of certain thermogenic genes in BAT (Xu et al., 2011; Ignacio et al., 2012), while another study showed a negative effect of 8weeks of treadmill training on the BAT biology with a decrease in BAT mass and the gene expression of Ucp1 and Pgc-1a (Wu et al., 2014). In humans, direct evidence of the effect of physical exercise on BAT function is very limited. Interestingly, endurance-trained athletes show significantly decreased cold-stimulated BAT activity compared to sedentary counterparts (Vosselman et al., 2015). Furthermore, short-term exercise training, such as high-intensity interval training and moderate-intensity continuous training, decreases insulin-stimulated glucose uptake in BAT in the individuals, who display highly active BAT prior to exercise (Motiani et al., 2017). The reduction in human BAT activation after above modes of exercises may be due to the exercise protocols or the involvement of other unknown mechanisms. Thus, further investigations on exercise-related adaption in human BAT function are required.

### Potential Mechanisms Involved in the Management of Oxidative Stress by Brown and Beige Adipose Tissues

**Figure 2:**
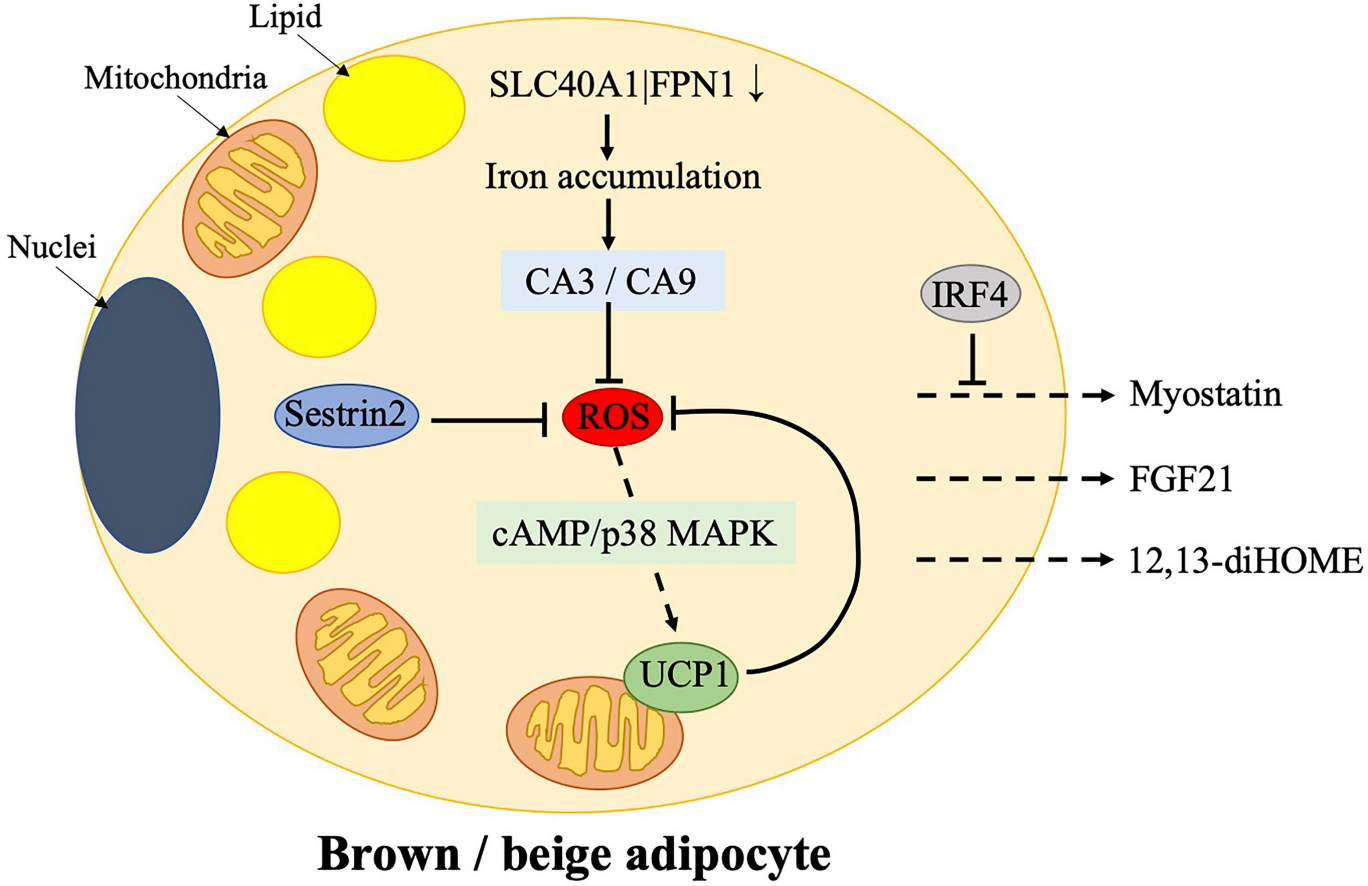
Mechanisms of ROS control in brown/beige adipocytes and the release of certain batokines, which target skeletal muscle. Physiological ROS enhances cAMP-p38 mitogen-activated protein kinase (MAPK) signaling to induce UCP1 expression in brown adipose tissue (BAT). UCP1, in turn, controls ROS production. Sestrin2, which is a stress-inducible protein, works as an antioxidant factor in BAT. However, over suppression of ROS by Sestrin2 results in an impaired upregulation of UCP1 after cold stimulation. Furthermore, ferroportin gene SLC40A1|FPN1, which mediates iron egress from cells, is downregulated in beige adipocytes, resulting in an iron accumulation. It is accompanied by an increased expression of carbonic anhydrases, CA3 and CA9, which mediate the protective effects on oxidative stress. BAT controls skeletal muscle function *via* the secretion of myostatin, which could be possibly prevented by IRF4. Additionally, 12,13-diHOME and FGF21, which are released from BAT, could also target skeletal muscles. CA, carbonic anhydrase; cAMP, cyclic adenosine monophosphate; 12,13-diHOME, 12,13-dihydroxy-9Z-octadecenoic acid; FGF21, fibroblast growth factor 21; IRF4, interferon regulatory factor 4; p38 MAPK, p38 mitogen-activated protein kinase; ROS, reactive oxygen species; and UCP1, uncoupling protein 1.

Several studies show that brown/beige adipose tissues may play a role in the management of oxidative stress, as shown in Figure 2.

#### Uncoupling Proteins

The uncoupling of mitochondrial respiration has been long found to protect from oxidative stress, because UCPs which uncouple the mitochondrial respiration are involved in controlling the ROS production in mitochondria. UCP2 and UCP3, which mainly express in the heart, protect heart from oxidative damage (Teshima et al., 2003; Ozcan et al., 2013; Perrino et al., 2013), while UCP4 and UCP5, which are mainly but not limited expressed in brain, display a neuroprotective effect probably through the inhibition of ROS production (Ivanova et al., 2010; Kwok et al., 2010). UCP1 is a thermogenic adipose tissue specific protein, which is located on the mitochondria inner membrane. In BAT, UCP1 plays a role in controlling ROS production and defense against oxidative stress (Dlaskova et al., 2010; Oelkrug et al., 2010). Mechanisms of UCP1-mediated ROS defense may include diminishing the probability of “reverse uncoupled respiration,” lowering mitochondrial hyperpolarization or UCP1 activation by oxidative stress products, such as hydroxynonenal (Echtay et al., 2003; Hoerter et al., 2004). Though, some studies doubt the necessity of UCP1 in the above process (Shabalina et al., 2006, 2014). Interestingly, forced expression of UCP1 in heart-derived H9C2 cells confers resistance to hypoxia/reoxygenation, and its over-expression in mouse heart protects from ischemic-reperfusion damage (Bienengraeber et al., 2003; Hoerter et al., 2004). Remarkably, UCP1 was also found in renal tubular epithelial cells, inhibiting mitochondrial ROS production and protecting from acute kidney injury (Jia et al., 2019).

Notably, skeletal muscle-specific UCP1 transgenic mice exhibit an anti-aging phenotype, displaying decreased atherosclerosis and obesity and prolonged life span (Klaus et al., 2005; Gates et al., 2007; Adjeitey et al., 2013). Mechanisms include an enhanced activity of catalase and SOD and an increased glutathione content, all of which have antioxidant effects (Adjeitey et al., 2013; Keipert et al., 2013). Besides, genes involved in spermidine synthesis, serine/glycine synthesis, and glutathione synthesis are upregulated in these transgenic mice as well, which are probably involved in the resistance to oxidative stress as well (Ost et al., 2015). Importantly, both mild oxidative stress and severe oxidative stress result in the activation of nuclear factor erythroid 2-related factor 2 (Nrf2; involvement of mitochondrial ROS; Muthusamy et al., 2012; Zucker et al., 2014), which promotes mitochondrial biogenesis and possibly plays a role in increasing endurance capacity (Ryoo and Kwak, 2018). The antioxidant effects of forced expression of UCP1 in skeletal muscle has been found to be Nrf2 dependent (Coleman et al., 2018). However, in certain pathological conditions, such as diabetes and obesity, Nrf2 activity is impaired (Rabbani et al., 2019), which could be restored by mitochondrial ROS elimination (Kasai et al., 2020). Thus, above findings indicate the physiological role of mitochondrial ROS and Nrf2 in mediating the healthy effect of exercise, and the mechanisms of UCP1-mediated antioxidant effects in skeletal muscles are probably Nrf2-related.

#### Iron Accumulation

A study on single-cell transcriptomes of adipose mesenchymal progenitors has revealed that certain subtype of adipocytes protects cells from oxidative stress (Min et al., 2019). Adipose tissues from neck or abdominal depots were obtained from humans, and large number of mesenchymal progenitor cells from a single cell were prepared according to their elaborate protocol (Min et al., 2016; Tran et al., 2018). Four distinct adipocyte subtypes with distinct expression of transcriptional factors and metabolic functions were identified. They look morphologically different and show different response to forskolin (Fsk), which usually stimulates the lipolysis. Among them, one cluster shows strong response to Fsk stimulation, expressing higher levels of thermogenic genes DIO2 and cell death-inducing DFF45-like effector C (CIDEC), but not UCP1, compared to the other clusters. Furthermore, expression of genes enriched in the ferroptosis and mineral absorption pathways is decreased in this cell type. Among these genes, the ferroportin gene SLC40A1|FPN1, which is the only mechanism known to mediate iron egress from cells, is downregulated. This results in an increase in iron accumulation. Iron is a rate-limiting and regulatory factor in adipose tissue browning and mitochondrial respiration, which shows close relation to redox stress (Kusminski et al., 2014). Subsequently, iron accumulation is accompanied by an increased expression of carbonic anhydrases (CAs), CA3 and CA9, which potently mediate the protective effects on oxidative stress. All the above findings in this type of thermogenic cells reveal a characteristic of rapid mitochondrial biogenesis and enhanced respiratory flux of the human beige adipocytes, suggesting a potential role of human beige adipocytes in the control of oxidative stress.

### White Adipose Tissue Impacts Skeletal Muscle Function

White adipose tissue is not only a depot of energy but also an active endocrine organ. WAT-derived adipokines such as adiponectin and leptin play important roles in the whole-body energy homeostasis. In particularly, chronic exercises have been shown to induce plasma levels of adiponectin and the expression of its receptors in the skeletal muscles in rodents as well as in humans (Bluher et al., 2006; Inoue et al., 2017). On the other hand, WAT influences skeletal muscle function through the secretion of above adipokines. Adiponectin displays anti-oxidative, anti-inflammation, and pro-myogenic effects on skeletal muscles as reviewed by Abou-Samra et al. (2020). It induces fatty acid oxidation through AMP protein kinase (AMPK)/acetyl-CoA carboxylase (ACC) signaling, mitochondrial biogenesis through AMPK/PGC-1a signaling, glucose uptake through the translocation of glucose transporter 4 to plasma membrane, inhibits oxidative stress by repressing nuclear factor-kappaB (NF-κB; involvement of AMPK), promotes muscle regeneration through multiple mechanisms including an induction of myogenic transcription factor expression such as myogenic differentiation 1 (MyoD; Li et al., 2011; Krause et al., 2019; Abou-Samra et al., 2020). However, leptin level is lowered in human plasma after exercise (Fedewa et al., 2018). Leptin has been found to induce glucose utilization in skeletal muscle (Toda et al., 2013), and promotes myogenesis *via* PGC-1a (Rodriguez et al., 2015, 2020a). Other adipokines such as retinol-binding protein 4, visfatin, vaspin, chemerin, or omentin are involved in the regulation of insulin sensitivity by the control of glucose uptake and fatty acid oxidation in skeletal muscle (Kahn et al., 2019). In consistence with the findings as mentioned previously, trained subcutaneous WAT transplantation in sedentary mice results in an improvement of glucose uptake into oxidative skeletal muscle, probably due to the altered adipokine profile in the trained WAT and the release of adipokines into the circulation (Stanford et al., 2015). Thus, exercise not only triggers the release of myokines from skeletal muscle but also stimulates the release of adipokines from WAT, which improve skeletal muscle function, as shown in Figure 1.

### Brown Adipose Tissue Affects Skeletal Muscle Function

Myostatin is known to be secreted by skeletal muscles and inhibits muscle function (Rodriguez et al., 2014). It has been previously mentioned that myostatin negatively regulates beige adipose tissue development, which is one of the mechanisms involved in BAT-skeletal muscle crosstalk. It has also been demonstrated that BAT controls skeletal muscle function *via* the secretion of myostatin (Kong et al., 2018). It has been found that interferon regulatory factor 4 (IRF4) in BAT, which was previously identified as a regulator for adipogenesis by the same group (Eguchi et al., 2008), contributes to exercise capacity and mitochondrial function in muscle. Specific loss of IRF4 in BAT induces myogenic gene expression in BAT and myostatin secretion from BAT. The induced myostatin in serum then influences the exercise capacity by impacting the function of white vastus muscle. On the contrary, mice with IRF4 over-expression in BAT shows a thermogenic phenotype, leading to an increase in ADP-stimulated and maximal mitochondrial respiration rates and mitochondrial DNA content in vastus lateralis, an improved running ability as well as a reduction in serum myostatin in mice. Furthermore, thermoneutrality results in a similar muscle phynotype as IRF4 knockout and an induced expression of myostatin in murine BAT as well. Thus, it reveals that BAT secrets myostatin under certain conditions, mediating a negative effect on skeletal muscle function, which is another mechanism involved in BAT-skeletal muscle crosstalk. Another batokine FGF21 appears to mediate the formation of cristae in mitochondria in skeletal muscle and BAT in cold environment (Bal et al., 2017). Additionally, a BAT-released lipokine, 12,13-dihydroxy-9Z-octadecenoic acid (12,13-diHOME), has been discovered in the circulation in humans after a bout of moderate-intensity exercise and in rodents following similar exercise protocol (Stanford et al., 2018). It induces fatty acid uptake and oxidation in skeletal muscles, which acts as a metabolic adaptation to a high exercise activity and may also be involved in the regulatory mechanisms of BAT-skeletal muscle crosstalk.

### ROS Sustain the Function of Thermogenic Fat

Reactive oxygen species accumulation is deleterious for BAT metabolism, which is one of the mechanisms involved in aging or obesity related BAT dysfunction (Shimizu et al., 2014; Graja and Schulz, 2015). Despite negative effects of ROS, they are considered to be important signaling molecules. Physiological ROS have been demonstrated to enhance the cyclic adenosine monophosphate (cAMP)/p38 mitogen-activated protein kinase (MAPK) signaling to induce the UCP1 expression in murine BAT (Ro et al., 2014). Sestrin2, which is a stress-inducible protein (Lee et al., 2012), plays a role in this process. However, overexpression of Sestrin2 reduces ROS accumulation in mice, which results in an impaired upregulation of UCP1 in BAT after cold stimulation. Furthermore, antioxidants cause an inhibition in cold or cAMP-induced UCP1 expression in murine BAT as well. These suggest that over suppression of ROS production is detrimental for BAT metabolism. Thus, they conclude that a physiological level of ROS benefits to a healthy BAT metabolism and adipose tissue physiology (Ro et al., 2015). It is believable that moderate exercises, which produce moderate levels of ROS, should be profitable for human health at least in this respect.

## Summary and Prospectives

Adipose tissues adapt to physical exercise in many aspects (shown in Figure 1) including (1) fat mobilization, which results in an induction of fatty acids to be up taken by skeletal muscle; (2) release of various adipokines from exercised WAT, which benefit skeletal muscle function in many aspects; (3) beige fat development, which is probably mediated by the release of various myokines from contracting muscle; and (4) promotion of BAT function, which is still controversial. Physical exercise also induces ROS production in human bodies. Depending on the intensity and duration of exercise, it elevates ROS with different levels (Thirupathi et al., 2020). Importantly, ROS participate in the regulatory mechanisms of some metabolic pathways, and are even essential for certain metabolisms. On the other hand, excessive ROS may cause cell, tissue, and organ dysfunction, which is one of the mechanisms involved in diseases including obesity, aging, etc. A moderate control of ROS production may lead to a healthier metabolism.

**Figure 3:**
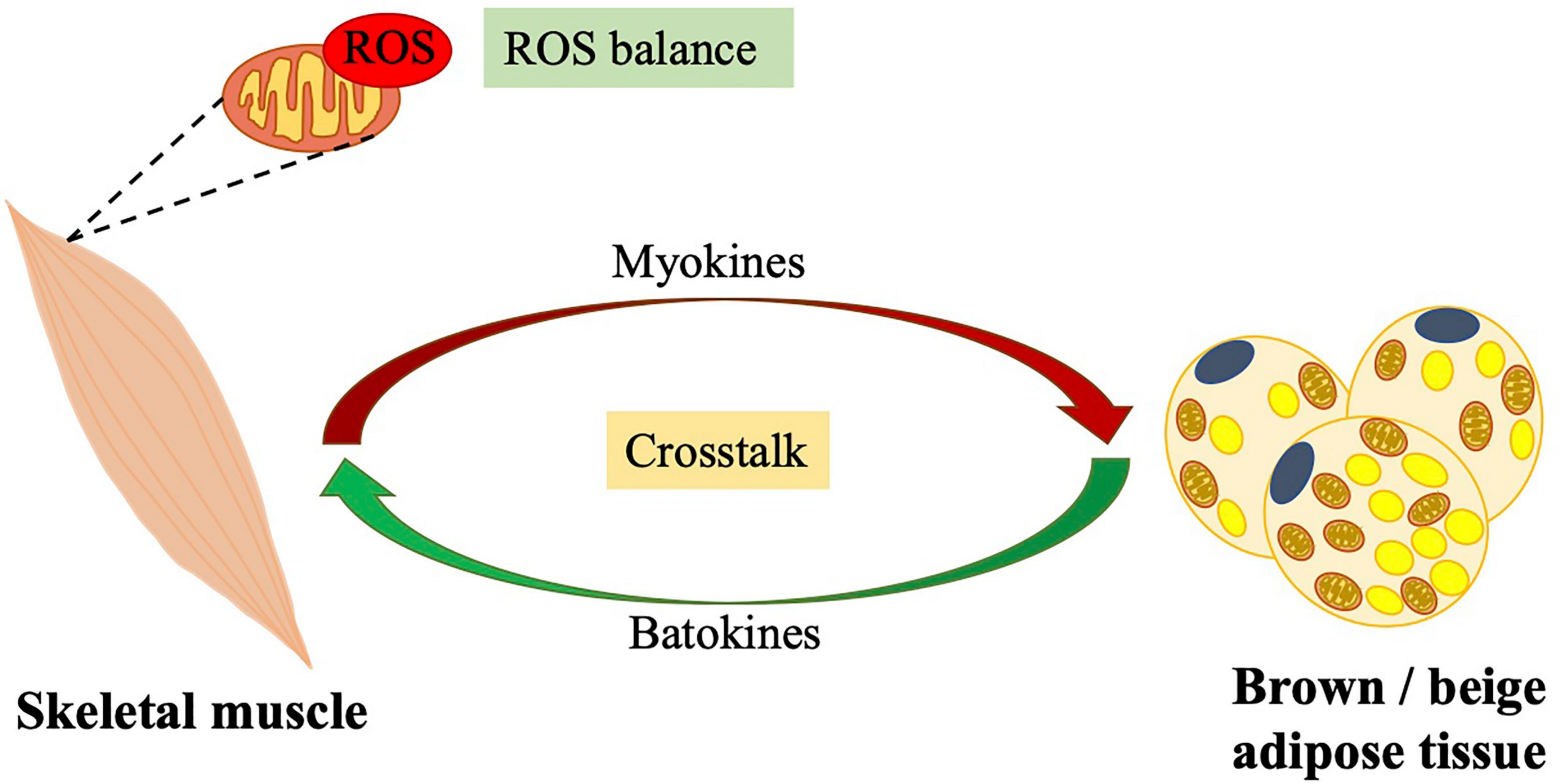
Crosstalk between skeletal muscle and brown/beige adipose tissues: importance of the balance of ROS levels. Contracting skeletal muscles produce a large amount of ROS. A cross talk between skeletal muscle and brown/beige adipose tissue, that certain batokines secreted from brown/beige adipose tissue control muscle function and various myokines secreted from contracting muscles promotes brown/beige adipose tissue function, may contribute to exercise-related ROS balance. ROS, reactive oxygen species.

Antioxidant enzymes are induced by oxidative stress after exercises, which is one of the known mechanisms involved in the antioxidant effects during exercise. Of note, there exist a crosstalk between skeletal muscle and thermogenic adipose tissues as described previously. Thermogenic adipose tissues benefit whole-body energy equilibrium through non-shivering thermogenesis, a process of heat generation accompanied by fat burning. Exercise induces the beige adipose tissue development and may promote BAT function *via* secretion of various myokines mostly according to rodent data. Additionally, thermogenic adipose tissues have been demonstrated to be involved in the antioxidative defense through mechanisms involving UCP1 and iron accumulation, as shown in section Potential Mechanisms Involved in the Management of Oxidative Stress by Brown and Beige Adipose Tissues and Figure 2. On the other hand, the batokines released from BAT, such as myostatin, FGF21 and 12,13-diHOME, impacts the function of skeletal muscle. The former inhibits muscle function; however, its secretion is suppressed by IRF4 in BAT. The latter promotes fatty acid uptake and oxidation in skeletal muscles. We presume that this crosstalk between skeletal muscle and thermogenic adipose tissues may be another mechanism involved in defense of exercise-induced oxidative stress and retain of a ROS balance, as shown in Figure 3.

Direct evidence on exercise-induced thermogenic adipose tissue function in humans is very limited. Nevertheless, as mentioned previously, current human studies show no beneficial effect of exercises on BAT metabolism. On the contrary, endurance-training, high-intensity interval training, and moderate-intensity continuous training decreased cold-stimulated BAT activity in humans. It indicates that other unknown mechanisms may be involved in exercise-caused changes in BAT function. Likely, the reduction in human BAT activation after above modes of exercises may be due to the exercise protocols, which are followed in the studies, suggesting that excessive ROS levels are possibly detrimental for BAT metabolism. Further investigation on the effects of exercise on BAT function in humans are thus required. Probably, the effect of Aerobic intermittent training should be studied to learn its effect on BAT function, since it has been shown to have protective effects on various diseases in humans.

Notably, some groups have identified possible mechanisms involved in mediating the beneficial effects of ROS on BAT metabolism as described in section Brown Adipose Tissue Affects Skeletal Muscle Function. It is indicated that physiological ROS levels are essential for a healthy BAT metabolism and thereby energy balance. Thus, either total suppression of ROS production or over production of ROS is not desirable. The control of accurate ROS levels, which requires a balance of ROS production and elimination, is however hardly accomplished by moderate exercise. A balanced physical exercise is good for a balanced whole-body metabolism. This review demonstrates reciprocal influences between skeletal muscle and thermogenic adipose tissues, contributing to the knowledge on the roles of brown and beige adipose tissues in the management of exercise-induced oxidative stress.

## Author Contributions

RP wrote the manuscript. YC revised the manuscript. All authors contributed to the article and approved the submitted version.

## Funding

This work was supported by a grant from National Natural Science Foundation of China (grant number 82070859 to YC and RP) and a grant from Tongji Hospital in Huazhong University of Science and Technology (grant number 2201103295 to YC).

## Conflict of Interest

The authors declare that the research was conducted in the absence of any commercial or financial relationships that could be construed as a potential conflict of interest.

## Publisher’s Note

All claims expressed in this article are solely those of the authors and do not necessarily represent those of their affiliated organizations, or those of the publisher, the editors and the reviewers. Any product that may be evaluated in this article, or claim that may be made by its manufacturer, is not guaranteed or endorsed by the publisher.
